# Association between Liver Cirrhosis and Diabetes Mellitus: A Review on Hepatic Outcomes

**DOI:** 10.3390/jcm10020262

**Published:** 2021-01-12

**Authors:** Laura I. Coman, Oana A. Coman, Ioana A. Bădărău, Horia Păunescu, Mihai Ciocîrlan

**Affiliations:** 1 Faculty of Medicine, “Carol Davila” University of Medicine and Pharmacy, 030167 Bucharest, Romania; laura.coman21@yahoo.com (L.I.C.); andreia.coman@gmail.com (O.A.C.); anca.badarau@umfcd.ro (I.A.B.); ciocirlanm@yahoo.com (M.C.); 2 Department of Gastroenterology, “Prof. Dr. Agrippa Ionescu” Clinical Emergency Hospital, 011356 Bucharest, Romania

**Keywords:** liver cirrhosis, diabetes mellitus, hepatogenous diabetes, hepatic encephalopathy, variceal hemorrhage, hepatocellular carcinoma, liver transplant

## Abstract

Background: Liver cirrhosis (LC) is largely associated with diabetes mellitus (DM). More than 80% of patients with LC manifest glucose intolerance and about 30% have type 2 DM. A particular and yet unrecognized entity is hepatogenous diabetes (HD), defined as impaired glucose regulation caused by altered liver function following LC. Numerous studies have shown that DM could negatively influence liver-related outcomes. Aim: We aimed to investigate whether patients with LC and DM are at higher risk for hepatic encephalopathy (HE), variceal hemorrhage (VH), infections and hepatocellular carcinoma (HCC). The impact of DM on liver transplant (LT) outcomes was also addressed. Methods: Literature search was performed in PubMed, Ovid, and Elsevier databases. Population-based observational studies reporting liver outcomes in patients with LC were included. Results: Diabetics are at higher risk for HE, including post-transjugular intrahepatic portosystemic shunt HE. DM also increases the risk of VH and contributes to elevated portal pressure and variceal re-bleeding, while uncontrolled DM is associated with increased risk of bacterial infections. DM also increases the risk of HCC and contributes to adverse LT outcomes. Conclusions: Patients with DM and LC may benefit from close follow-up in order to reduce readmissions and mortality. Due to the heterogeneity of available research, prospective multicenter clinical trials are needed to further validate these findings.

## 1. Introduction

Type 2 diabetes mellitus (T2DM), a disorder of glucidic metabolism mainly accompanied by hyperglycemia and insulin resistance (IR), is largely associated with liver cirrhosis (LC). More than 80% of patients with LC manifest glucose intolerance and approximately 30% have T2DM [1,2]. Type 1 diabetes mellitus (T1DM), an insulino-dependent disease, might also involve hepatic dysfunction [3,4].

Liver involvement in T2DM is well recognized in the form of non-alcoholic fatty liver disease/metabolic associated fatty liver disease (NAFLD/MAFLD), which can range from simple steatosis to non-alcoholic steatohepatitis (NASH) and LC, with or without hepatocellular carcinoma (HCC) or liver failure as indications for liver transplantation (LT) [5].

MAFLD is a new interpretation of the old NAFLD and is defined by the presence of hepatic steatosis accompanied by each of the following: (1) overweight or obesity, (2) T2DM or (3) normal weight with evidence of metabolic dysregulation (prediabetes, IR, inflammation, decreased HDL-cholesterol levels or elevated triglyceride levels). Although the pathophysiology of NAFLD and MAFLD is the same, in subjects with metabolic dysregulation, the term MAFLD seems to be more appropriate and brings fatty liver disease closer to T2DM [6,7].

Meanwhile, poorly controlled T1DM can lead to rare complications like glycogenic hepatopathy (formerly Mauriac syndrome) [3] and diabetic hepatosclerosis, recently described as a noncirrhotic form of hepatic microangiopathy of the liver [4].

Besides T1 and T2DM, hepatogenous diabetes (HD) is a recently described and yet unrecognized clinical entity with particular physiopathological mechanisms and systemic complications. It can be defined as a state of impaired glucose regulation caused by altered liver function following LC [8]. It typically presents with hyperinsulinemia, normal fasting plasma glucose (FPG) and glycosylated hemoglobin (HbA1c), but abnormal response to oral glucose tolerance test (OGTT) [9].

Through a variety of mechanisms, LC clearly contributes to dysglycemia by interfering with the insulin-glucose metabolism, while DM predisposes to liver disease progression and a higher risk of serious complications of LC, presuming that patients with compensated LC and concomitant DM may be at greater risk of decompensating events [10].

T2DM, in particular, is associated with adverse outcomes in patients with LC, including higher rate of hospital admissions, higher prevalence of HCC, and increased risk of mortality [11].

Like T2DM, HD might also be associated with increased rate of complications [12]. However, the clinical impact of HD on LC has yet to be elucidated, given that the prognosis of patients who develop HD is more likely to be negatively affected by the underlying hepatic disease and its complications than by HD itself [13]. In our review, we considered HD as a particular type of DM, along with T2DM and T1DM, and addressed it separately only where it was described as a distinct entity.

Numerous studies have shown that DM could negatively influence liver-related outcomes [10,11,14]. However, a more systematic approach is needed to confirm this association and to determine if diabetic patients with LC may benefit from a closer follow-up.

We conducted a review aiming to investigate whether patients with LC and DM are at higher risk for complications of LC. Additionally, we aimed to provide an overview on the available evidence regarding the impact of DM on LC complications and to formulate further research possibilities.

We addressed well-known complications of LC, i.e., hepatic encephalopathy (HE), variceal hemorrhage (VH), infections, and hepato-renal syndrome. Further, we performed an update regarding the impact of DM on HCC and LT, according to the current available research.

## 2. Materials and Methods

A literature search was performed by two independent authors of the review in three databases. We searched for reviews and clinical studies performed on humans in the last 10 years.

The date of the first literature search was 03.11.2020, in the PubMed database, followed by a second and a third, both in the Ovid database, in 04.10.2020 and 09.10.2020, respectively.

The first step of selecting the studies consisted in a review of titles and abstracts by two reviewers. If there was a disagreement, the full text was obtained and a second screening was performed until final agreement was reached.

**Figure 1:**
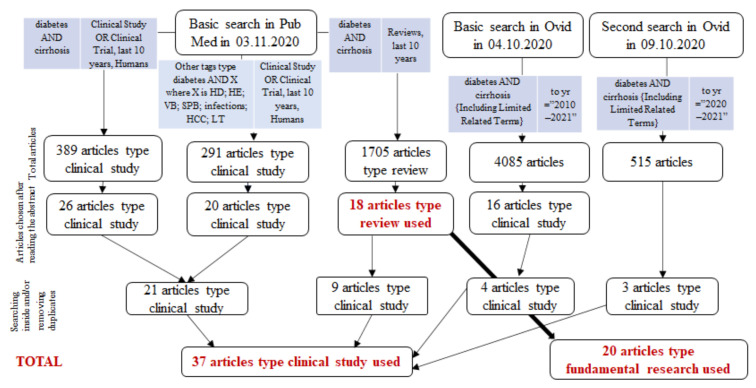
Diagram representing research methodology flowchart. Legend: HD = hepatogenous diabetes; HE = hepatic encephalopathy; VB = variceal bleeding; SBP = spontaneous bacterial peritonitis; HCC = hepatocellular carcinoma; LT = liver transplant.

We included population-based observational studies that reported liver disease outcomes in patients with already diagnosed LC of any severity. Studies assessing only HCC as an outcome in the context of a non-cirrhotic liver were also eventually included and compared. The results of this literature search are presented in Figure 1. Case reports and articles that emphasized possible treatments were excluded.

Additional literature search in PubMed, Ovid and Elsevier Science Direct was performed in 12.15.2020 and revealed another 7 records, including two clinical studies. Research methodology for this additional literature research was not included in Figure 1.

Studies regarding the impact of DM on hepatorenal syndrome were actively searched, but no significant clinical research was found.

## 3. Results

### 3.1. Hepatogenous Diabetes as a Complication of Liver Cirrhosis

HD is defined as the development of dysglycemia and impaired insulin regulation following long-term evolution of LC. More specifically, it is believed to be a result of altered liver function, impaired insulin clearance, and pancreatic β-cell dysfunction, leading to hyperinsulinemia and defective use of blood glucose [12].

HD typically develops as a complication of LC in patients without common risk factors for T2DM: high body mass index (BMI), family history of DM and hyperlipidemia. Conversely, T2DM may be diagnosed before LC or simultaneously (especially in patients with NASH cirrhosis), with traditional risk factors playing a major role [15]. However, in clinical practice, discrimination between HD and T2DM may be difficult or even impossible and, despite having distinct pathophysiological and clinical implications, the American Diabetes Association does not recognize HD [16].

Moreover, HD seems to be significantly correlated with poor prognosis and decreased 5-year survival rate [13].

Diagnostic criteria for HD are the same as for primary DM, with some particularities [1]. OGTT seems to be the most accurate screening test for HD, given that FPG can be normal or even decreased early in the disease, especially in patients with advanced LC. Furthermore, the evaluation of fasting plasma insulin (FPI) and homeostatic model assessment of insulin resistance (HOMA-IR) could predict the early development of IR. HbA1c, however, is less accurate compared to OGTT for the diagnosis of HD in patients with LC associated with moderate to severe anemia [17].

A study evaluating the prevalence of glucose metabolism disorders (GMDs) in adult patients with compensated LC showed that almost 80% out of a total of 130 had GMDs, half of them being subclinical. They used, for the diagnosis, FPG, FPI, and OGTT, and the HOMA-IR index, for the detection of IR. According to the test results and chronology regarding the diagnosis of DM and LC, GMDs were classified as T2DM, HD, and impaired glucose tolerance (IGT). As a result, T2DM was diagnosed in 25 patients (19.2%, 95% CI: 12.5–25.9), HD in 28 (21.5%, 95% CI: 14.5–28.5), and IGT in 36 (38.5%, 95% CI: 30.1–46.7), while 48.7% of the total had IR [2].

### 3.2. Diabetes Mellitus and Hepatic Encephalopathy

Numerous studies have linked LC-associated DM with an increased incidence of hepatic encephalopathy (HE) and an overall increase in the likelihood of decompensation from HE.

Additional information is provided in Table S1. One of the first studies to investigate the association between DM and HE was performed in patients with hepatitis C virus (HCV)-related LC. DM was independently associated with severity of HE (*p* = 0.007), while patients with DM had severe HE at an earlier stage of biochemical decompensation of LC that non-diabetics [18].

A prospective study investigating the effect of DM on prevalence and severity of HE in patients with decompensated LC concluded that cirrhotic patients with DM were more likely to present HE; the severity of HE was also greater in patients with DM (*p* = 0.01). Moreover, older age (>60 y), along with DM, independently increased the prevalence of HE (*p* = 0.03 and 0.006, respectively) [19].

Another study conducted by Elkrief L. et al., 2014 provides further evidence that DM could be a risk factor for complications in patients with HCV-related LC, being associated with HE at inclusion (*p* < 0.001) and resulting in shorter LT-free survival in patients with model for end-stage liver disease score (MELD) <10 at baseline (*p* < 0.005) [20].

To further investigate DM as a risk factor for overt HE, Jepsen P. et al., 2015 analyzed data from 862 patients with LC and ascites included in three multinational randomized controlled trials of satavaptan. Although precipitating factors for HE did not differ between diabetics and non-diabetics (*p* = 0.84) and fewer diabetics than non-diabetics were in Child-Pugh class C at baseline (13% vs. 23%), diabetics had higher 1-year cumulative risk of first-time overt HE (26.0% vs. 15.8%), and their episodes of first-time overt HE were more likely to progress beyond grade 2. Furthermore, blood glucose was not correlated with HE; the rate of first-time overt HE was not higher in periods of hyperglycemia than in periods of euglycemia (aHR = 1.14, 95% CI 0.49 to 2.64) [21].

Liu T-L. et al. examined the risk of decompensation in patients with LC and DM and found that HE was more prevalent in diabetics: 15.35% vs. 13.26% in non-diabetics [10].

Yin X. et al. found that DM significantly increased the incidence of first-time overt HE within 12 months after creation of transjugular intrahepatic portosystemic shunt (TIPS) (*p* = 0.026). More exactly, DM (*p* = 0.015) and older age (*p* = 0.002) were independent predictors for HE after TIPS [22]. Routhu M. et al. also found that DM was a predictor of HE after TIPS [23].

Another recent study revealed prospective evidence for the association between DM, covert HE (as defined by the Psychometric Hepatic Encephalopathy Score), and overt HE. They found that poor glycemic control (HbA1c ≥ 6.5%) was strongly associated with the development of both covert HE and overt HE. DM was independently associated with the development of overt HE during follow-up, both in patients with previous overt HE (HR = 1.976; 95% CI: 1.286–3.038, *p* = 0.0268) and in patients without history of overt HE (aHR 3.23, 95% CI: 1.34–7.76; *p* = 0.009). Thus, DM increased the risk of first-time overt HE by up to 76% (aHR = 3.23), although DM patients had better liver function than non-diabetics [24].

### 3.3. Diabetes Mellitus and Variceal Hemorrhage

Research on how DM influences the risk of variceal hemorrhage (VH) in patients with LC is limited.

Additional information is provided in Table S2. A prospective study aimed to assess the prevalence of HD and its impact on portal pressure and VH. HD was diagnosed in 55.4% of patients with LC without history of DM; among them 62% had normal FPG, but impaired OGTT, consistent with the definition of HD. HD showed significant correlation with Child-Pugh score, VH, and hepatic venous pressure gradient (HVPG) (*p* = 0.004, *p* = 0.002, and *p* = 0.019, respectively). More specifically, higher 120-min glucose levels during OGTT were significantly correlated with increased HVPG and incidence of VH (*p* < 0.001, p = 0.042, respectively). IR was also correlated with increased HVPG (*p* = 0.011), but not with VH (*p* = 0.958).

More than a half of the non-diabetic cirrhotic patients actually had GMDs in the form of HD, (as defined by the above mentioned criteria), with more than 60% having normal FPG, which was therefore not a predictor of VH (*p* = 0.553). OGTT was superior for evaluation of GMDs in cirrhotic patients without history of T2DM, while 120-min glucose levels and IR positively correlated with increased HVPG [25].

Besides HD, T2DM is an independent risk factor for VH among LC patients. Yang CH. et al. showed that diabetic patients had higher ratio of Child-Pugh Class B/C (*p* = 0.043), renal insufficiency (*p* = 0.002), and history of VH (*p* = 0.006) than non-diabetics. Moreover, T2DM and Child-Pugh class B/C were identified as independent predictors of VH, in a subgroup analysis, T2DM significantly correlated with VH only in patients with Child-Pugh class A (*p* = 0.042) [26].

Additionally, a previous study also showed that IR, regardless of the presence of DM, significantly predicted the presence of esophageal varices in chronic hepatitis C patients with Child-Pugh class A LC [27].

A prospective case-control study on the effects of DM on clinical course and mortality of acute VH in 60 cirrhotic patients showed that diabetics had significantly more unstable course, many hospital admissions during follow-up, and higher mortality rate than non-diabetics. Most importantly, diabetics had higher incidence of re-bleeding (46.4% vs. 10%) as well as higher incidence of HE (36.7% vs. 10%), although the 2 groups had similar functional liver status. Moreover, diabetics had higher rate of hepatosplenomegaly and more portal vein dilations and collaterals, assuming they had increased portal pressure [28].

### 3.4. Diabetes Mellitus and Infectious Complications of Liver Cirrhosis

Spontaneous bacterial peritonitis (SBP) is the most frequent type of infection in individuals with decompensated LC. A prospective, case control study on 475 patients analyzed the impact of T2DM and HbA1c values on the incidence of SBP and mortality in patients with LC and ascites. In conclusion, patients with T2DM had increased risk for developing SBP and SBP incidence was significantly higher in patients with HbA1c values ≥6.4%. Additionally, DM and HbA1c values ≥6.4% were significantly associated with higher incidence of recurrent SBP episodes (*p* = 0.04 and *p* = 0.02, respectively). Interestingly, T2DM patients with HbA1c < 6.4% at baseline had a similar risk for SBP as those without T2DM [29].

Additional information is provided in Table S3. LC is also a strong risk factor for pyogenic liver abscess (PLA). The etiology of PLA is probably associated with structural changes in the cirrhotic liver including reduced trans-hepatic blood flow, increased portal pressure and often ascites. A retrospective case-control study conducted by Ko et al. showed that T2DM was significantly associated with increased risk of PLA, especially in male and young subjects (<45 years). T2DM patients with underlying biliary tract diseases and LC are at even greater risks of PLA [30].

A prospective case control study on a database of 3 randomized controlled multicentric trials investigating the efficacy of satavaptan in treating ascites compared the risk of infections and mortality following an infection between patients with LC, with or without DM. The study had conflicting results: DM did not increase the HR of infections or the mortality following an infection in any of the diabetic patients receiving different associated treatments: diuretics, quinolones, proton pump inhibitors, lactulose, and non-selective beta-blockers [31].

Some other studies showed a greater risk for developing urinary infections [32], SBP and others, while DM was independently associated with bacterial infections in cirrhotic patients with HCV [20].

A recent database study on a large cohort of patients with DM and LC (906,559) from a Nationwide Inpatient Sample, USA, found that uncontrolled DM was associated with an increased risk of bacterial infections (OR 1.33, 95% CI 1.29–1.37, *p* < 0.001). The presence of multiple infections was more common in patients with uncontrolled vs. controlled DM (9.0% vs. 6.3%, *p* < 0.001), urinary tract infections (UTI), pneumonia, cellulitis, and sepsis being the most prevalent (all *p* < 0.001). There was no significant relationship between uncontrolled DM and SBP, Clostridium difficile infection or cholangitis individually (all *p* > 0.20) [33].

### 3.5. Diabetes Mellitus and Hepatocellular Carcinoma

There are conflicting results of earlier studies regarding DM and HCC, and the evidence of a link between LC associated DM and HCC was illustrated in only a few of them.

A state-wide registry from Denmark analyzed between 1977 and 1993, consisting of 109,581 patients with DM, showed standardized incidence ratios for primary liver cancer of 4.0 in males and 2.1 in females. Standardized incidence ratios remained elevated with increasing years of follow-up and also after exclusion of patients with reported risk factors (e.g., LC and hepatitis) [34]. Moreover, a 2006 meta-analysis of retrospective cohort studies and case-control studies showed an association between T2DM and an increased risk of HCC [35].

Li Q et al. analyzed the role of T2DM in the development of HCC in patients with chronic hepatitis B. The prevalence of T2DM was higher among HCC patients without LC than among those with LC (12.1% vs. 6.7%, *p* = 0.003). Among female patients without LC, T2DM was strongly associated with HCC risk with OR = 5.6 (95% CI: 2.2–14.1) [36].

In a Chinese population with high prevalence of hepatitis B virus (HBV) infection, DM was found to be an independent risk factor for HCC. Age and male gender also had a positive correlation with HCC. Although the study proved that DM and LC are separate independent risk factors for HCC, the probability of developing HCC in patients with both DM and LC was not evaluated [37].

Furthermore, hyperinsulinemia was defined as an independent risk factor for HCC among HBV carriers. The effect of higher insulin on HCC risk persisted after adjustment for other metabolic factors, and was consistent across strata of age, BMI and HBV genotype being more profound among those with lower viral load <4.39 log_10_ copies/mL at recruitment (HR = 6.15, 95% CI: 2.48–15.22). The study was conducted in Taiwan (1989–2006) on a cohort of 2903 male government employees chronically infected with HBV [38].

In contrast, neither DM nor increased BMI were a risk factor for HCC in a dual HBV and HCV endemic area of southern Taiwan in community cross-sectional and case-control studies performed by Tung HD et al., 2010. Only male gender, age ≥65 years, presence of HBsAg and anti-HCV antibodies, thrombocytopenia, and high alanine aminotransferase (ALT) levels were independent risk factors for HCC [39]. Regarding thrombocytopenia, in Japanese T2DM patients with platelet counts <200 × 10^3^/µL and probable LC, standardized mortality rate of HCC increased from 3.57 to 6.58 (95% CI: 4.34–9.58). Therefore, thrombocytopenia could be a negative prognostic factor for HCC in T2DM [40].

A prospective cohort study showed that there was no association between DM and HCC in patients with HCV-related LC, but in patients without HCV, DM was significantly associated with the risk of developing HCC. The lack of association between DM and HCC was further demonstrated in 410 patients with HCV-related LC enrolled in the HALT-C trial. These results may indicate that in HCV patients who already have a very high risk of HCC, DM may not increase this risk any further [41].

In the USA, between 2001 and 2010, 150 veterans with HCC were analyzed and compared to frequency-matched (2:1) non-cancer controls. DM could not be confirmed as a major risk factor for HCC in the overall cohort and in LC patients. In a subgroup analysis of HCC patients without LC and other risk factors such as chronic hepatitis B, chronic hepatitis C, alcohol-related liver disease and hemochromatosis, DM has become a strong independent predictor of HCC [42].

Yang J.D., 2020, in a prospective cohort study on 354 patients with NASH cirrhosis, showed that the 5-year cumulative incidence rate of HCC was 7.8%: 10.2% for DM patients vs. 1.7% for non-diabetics. DM was associated with an increased risk of developing HCC in univariate (HR = 3.6, 95% CI: 1.1–11.9; *p* = 0.04) and multivariate analysis (HR = 4.2, 95% CI: 1.2–14.2; *p* = 0.02). Increasing age and low serum albumin were significantly associated with increased risk of developing HCC in multivariate analysis [43].

Interestingly, donor characteristics and liver graft quality could play a role in the prognosis and outcome of liver disease after LT. Liver grafts from donors with raised BMI, history of DM, and severe graft steatosis indicate a higher recurrence risk of HCC after transplantation, even after adjusting for tumor and recipient characteristics, underlying liver disease, time spent on waiting list, and transplant center [44].

A prospective case-control study within the European Prospective Investigation into Cancer and Nutrition, showed that higher circulating concentrations of IL-6, C-reactive protein (CRP), C peptide, non-high molecular weight (HMW) adiponectin, and glutamate dehydrogenase were significantly associated with higher risk of HCC, independent on established liver cancer risk factors and high BMI. Incidence rate ratios and 95% CIs for HCC were per doubling of concentrations of CRP, IL-6, C-peptide, and non-HMW adiponectin (1.22; 95% CI: 1.02–1.46; *p* = 0.03; 1.90; 95% CI: 1.30–2.77; *p* = 0.001; 2.25; 95% CI: 1.43–3.54; *p* = 0.0005; and 2.09; 95% CI: 1.19–3.67; *p* = 0.01, respectively). C-peptide, as a marker of hyperinsulinemia, was strongly associated with risk of HCC and intrahepatic bile duct cancer, even after adjusting for HBV/HCV infection and inflammation, giving support to the hypothesis that hyperinsulinemia may increase risk of HCC and intrahepatic bile duct cancer [45].

Table 1 presents the synthetic relationship between increased risk of HCC and presence of LC and DM. Other variables like age, gender, HBV/HCV infection, thrombocytopenia, BMI, and steatosis are mentioned. Apart from the heterogeneity of studies, DM was clearly the most important risk factor for HCC.

### 3.6. Diabetes Mellitus and Liver Transplant

There should be increased awareness regarding the impact of early GMDs, T2DM, and other factors like obesity or alcohol consumption on the progression of NASH to LC [46], given that NAFLD/MAFLD, along with cryptogenic cirrhosis, have become leading indications for LT in the US [47].

New onset DM after LT was precocious and more frequent in patients receiving LT after having LC complications (ascites, hepatic coma, and esophageal variceal disease). An interesting study retrospectively analyzed a large Taiwanese nationwide cohort of patients who had received LT between 1998 and 2012. New-onset DM after LT was diagnosed in 8.4% of study participants, with higher first year incidence (5.38%), compared to 4.31–6.38% incidence of DM in the whole population of Taiwan between 2000 and 2009, with 0.764–0.932% first year incidence, respectively [48].

A single-centered, retrospective study of 415 adult LT recipients (2003–2016) (13% of them being transplanted for NASH) showed that post-LT DM incidence was 34.7%, 46.9%, and 56.2% at 1, 3, and 5 years, respectively, with overall survival of 90%, 80.9%, and 71.7%, respectively, half of the cases developing by 6 months and 75% by 12 months. The post-LT DM group had more rejection episodes (31.9% vs. 21.8%) and worse 5-year survival rates, age being the only significant predictor of post-LT DM [49].

Another single-centered retrospective study of LT recipients (1990–2015) showed that the incidence of post-LT DM was higher during the first year (87%); on multivariate analysis, NAFLD/MAFLD and the use of tacrolimus and sirolimus were independently associated with post-LT DM development. The authors also found that pre-LT DM increased the risk of end-stage renal disease and major cardiovascular events and, contrary to other findings, patient survival was comparable to those with post-LT DM. Thus, understanding the impact of post-LT DM on survival and adverse outcomes would need prolonged follow-up [50].

## 4. Discussion

The presence of DM and LC in the same patient represents a double pathological insult for the liver, increasing the risk of decompensating events, morbidity, and mortality, regardless of the initial etiology of LC [10]. Overall, DM and other GMDs were associated with increased risk of HE, VH, infections, and HCC and showed negative impact on LT outcomes.

Some studies showed that diabetics developed their first overt HE episode at an earlier point in the clinical course of LC than non-diabetics, and had more severe first-time episodes of overt HE [18,19,21]. Clearly, the incidence of HE was greater in patients with LC associating DM. Sigal et al., 2006 showed that DM increased the risk of overt HE with 17% (*p* = 0.007) [18], while according to Butt et al., 2013, overt HE risk increased with 15.9% in DM patients with LC (*p* = 0.03) [19]. Jepsen et al., 2015, described a risk of overt HE of 26% in DM patients vs. 15.8% in non-DM patients after 1 year of follow-up [21]. Additionally, according to Yin et al., 2019, the first year incidence of HE after TIPS was 16.6% higher in DM patients [22]. Rothu et.al., 2017 also showed that diabetics were at higher risk of post-TIPS HE [23]. Ultimately, Labenz et al., 2020 found that cirrhotic patients with DM and previous overt HE had 7.7% more episodes of first-time overt HE during follow-up (*p* = 0.026), while those without a history of overt HE had 5.5% more episodes of first-time overt HE (*p* = 0.009), leading to an overall increase in the incidence of overt HE of 13.2% [24].

Particularly, patients with DM and poor glycemic control were found to be at higher risk for covert HE and showed higher cumulative overt-HE incidence [24]. In a previous study, hyperglycemia was not correlated with overt HE [21] and the other mentioned studies did not evaluate this correlation.

LC generates hyperinsulinemia by reduced hepatic insulin clearance due to porto-systemic shunting and loss of hepatocytes, while both LC and DM contribute independently to hyperinsulinemia and IR [51]. Therefore, in cirrhotic patients, hyperinsulinemia and IR could be related to HE even in the absence of hyperglycemia or altered FPG. Thus, limiting DM detection to only evaluating FPG or HbA1c may not be of clinical utility in this setting [17].

Further, IR promotes protein catabolism and consequently increases ammonia production [52]. However, there is currently no evidence of a link between serum ammonia and severity of HE. Ammonia crosses the blood-brain barrier (BBB) and is metabolized in astrocytes by glutamine synthetase to glutamine, resulting in astrocyte swelling and generation of reactive oxygen species, thereby contributing to the cerebral dysfunction seen in HE [53]. Similar to serum ammonia, hypo- or hyperglycemia may not be a predictor of overt HE in clinical practice [21], meaning that other mechanisms could interfere with the risk of overt HE in patients with DM and LC.

Both DM and IR are associated with inflammation by release of proinflammatory cytokines. More specifically, TNF-α and IL-6 are higher in patients with LC, increasing the severity of HE by alteration of the BBB and neurotransmission activity [54,55]. DM also predisposes to infections, which further enhances the release of proinflammatory cytokines [56]. In addition, autonomic dysfunction in DM contributes to prolonged duodenal-cecal transit time, promoting constipation, small intestinal bacterial overgrowth (SIBO), and bacterial translocation, resulting in increased intestinal ammonia production [18]. Dysbiosis, increased gut permeability, SIBO [57], and IR [54] are further associated with inflammation and bacterial translocation [58]. This should lead to development of early diagnostic methods and potential therapeutic interventions targeting SIBO in patients with DM who are at risk for HE.

Although the results of the studies provide evidence that DM is a risk factor for HE in patients with LC, independent on the status of liver impairment, there are some limitations. Many factors such as the presence of other decompensating events (e.g., VB, SBP), TIPS, and alcohol abuse could impact the severity of HE and should be considered, especially in diabetic patients. Moreover, exclusion criteria were not mentioned in all of the studies, although these should be applied before investigating HE. Advanced age, the use of lactulose and/or antibiotics or other medications could also be a confounder for the development of HE and patient subgroups should be adjusted accordingly.

Future research should try to elucidate the influence of glycemic control on HE risk in diabetic patients with LC and should further investigate the impact of DM on covert HE.

Regarding VH, analyzing the available research, Jeon H.K. et al., 2013, found that HVPG was significantly and linearly correlated with 120-min plasma glucose levels during OGTT (*p* < 0.001) and that more HD patients had large varices (>5 mm) (32.8% vs. 17.9%), and consequently significantly increased incidence of VH compared to non-HD patients (25.6% vs. 10.8%, *p* = 0.001) [25]. Yang CH. et al., 2014 showed that patients with LC and T2DM had significantly higher incidence of VH than non-diabetics (32% vs. 12%) [26]. Moreover, Khafaga et al., 2015 related that, after acute VH, diabetics had higher incidence of re-bleeding (46.4% vs. 10%) and higher mortality rate (16.6% vs. 6.7%) than non-diabetics [28].

Although DM affects arterial function and contributes to systemic arterial hypertension and, in particular, pulmonary hypertension [59], the effect on veins as part of diabetic vasculopathy is far from being proved. Hyperglycemia and especially fluctuating blood sugar levels seem to contribute to splanchnic hyperemia and elevated portal pressure, increasing the risk of VH [60,61]. More, there is an important link between IR/HOMA-IR and impaired portal hemodynamics [62,63] and some studies suggested that IR could predict the presence of esophageal varices (EV) with 75% sensitivity and 80% specificity [27,64].

HD, as a particular entity, seems to be related to portal pressure in a reciprocal way [12,65]. However, the exact physiopathological mechanism is still unknown and has yet to be elucidated.

Concluding evidence suggests that DM increased the risk of VH and predisposed to variceal re-bleeding and unstable clinical course, including higher mortality rate [28]. In particular, IR and fluctuating serum glucose seem to affect HVPG [12]. Thus, maintaining as much as possible an euglycemic state and managing postprandial hyperglycemia may reduce portal pressure variations, and consequently the risk of VH.

Apart from HE and VH, infections may appear as a complication of both DM and LC. In patients with advanced liver disease, common infections include SBP, urinary tract infections, pneumonia, dermatologic infections, and bacteraemia [66].

According to current research, Tergast et al., 2018 showed that diabetics had higher incidence of SBP than non-diabetics (20% vs. 13%). Moreover, diabetics with HbA1c values ≥6.4% had increased incidence of SBP compared to those with HbA1c values <6.4% (25% vs. 16%). While DM patients with HbA1c values <6.4% as well as non-DM patients had a 48% risk of developing SBP (HR = 0.93, *p* = 0.78), diabetics with HbA1c values ≥6.4% had an almost two-fold increased risk of developing SBP, more exactly 80% (HR = 4.21, *p* = 0.0002) [29]. Ko M.C. et al., 2019 showed that T2DM patients had a higher cumulative incidence of PLA than controls (0.87% vs. 0.30%), DM being associated with increased hazard of PLA (HR = 2.88) [30]. In contrast, Bossen L. et al. 2019, found no association between DM and risk of infection in patients with LC and ascites treated with satavaptan [31].

The etiology of infections in cirrhotic patients is probably associated with impaired immune defence and structural changes in the cirrhotic liver, including reduced trans-hepatic blood flow, impaired macrophage function, reduced number of Kupffer cells, and impaired functions of neutrophils. Moreover, both DM and LC contribute to immune dysfunction and increased intestinal permeability to bacteria, further leading to systemic inflammation and higher risk of infections [67]. DM, in particular, is associated with high risk of infections and glycemic control is of uppermost importance for prevention and also during an acute infected state [68].

Overall, patients with DM experienced higher incidence of SBP, especially those with HbA1c values ≥6.4% [29]. Uncontrolled DM was associated with increased risk of multiple bacterial infections (UTI, pneumonia, cellulitis, and sepsis being the most prevalent) [33], proving that tight glycemic control could lower the risk for developing infections in cirrhotic patients with DM. However, considering the small number of studies and the contradictory findings, future research regarding the risk of infections in patients with DM and LC is needed.

Furthermore, the complex relationship between DM, LC, and HCC has led to varying degrees of uncertainty so far, and the heterogeneity of prior and current studies still brings little clarity to it. Although DM was identified as a predisposing factor for HCC in many of the analyzed studies, not all of them showed a link between DM, LC, and HCC. Some studies showed that DM was linked to HCC as an independent risk factor [37,38,42].

Interestingly, DM, raised BMI, and severe steatosis in liver graft donors predisposed recipients with prior HCC to higher recurrence risk of HCC after LT [44]. Moreover, DM was related to increased risk of developing HCC in patients with NASH cirrhosis [40,43]. It is well known that DM and IR predispose to fatty liver disease and accelerate progression of hepatic fibrosis, thus indirectly contributing to hepatocarcinogenesis. Additionally, hyperinsulinemia may be an important risk factor for cancer [69] and plays a major role in increased incidence of neoplasia in diabetic patients [70]. Besides hyperinsulinemia, inflammation is often associated with development and progression of cancer [71] and seems to be positively related to increased risk of HCC [72].

More importantly, the last decade has witnessed a shift in HCC etiology: along with the emergence of antiviral eradication treatments for HCV infection, there is a significant increase in incidence of obesity, metabolic syndrome, DM, and NASH cirrhosis. Besides the increasing global prevalence and burden of MAFLD [73], it has become one of the most important non-viral causes of HCC and there is increasing evidence that HCC can develop even in noncirrhotic/MAFLD [74]. However, long-term prospective studies are needed in order to confirm this unfavorable consequence.

Although MAFLD and DM seem to be associated with a higher risk of HCC, strategies for screening are cost-effective only if the incidence of HCC reaches more than 1.5% per year. There is insufficient evidence at this moment regarding a recommendation of HCC surveillance in noncirrhotic MAFLD and recommendations are weak for noncirrhotic subjects with F3 fibrosis [75]. Moreover the techniques that should be used to screen MAFLD and DM patients for HCC are still debatable and there are future perspectives for personalized strategies based on risk stratification and molecular testing [76].

Regarding the association between DM and LT, new onset DM was more prevalent in patients receiving LT, compared to general population [15,48]. Overall, post-LT DM seemed to have higher first-year incidence and outcomes trended mostly towards increased rejection and worse survival among post-LT DM individuals, suggesting the benefit of early strategies targeting glucose control [77]. Pre-LT DM also increased the risk for post-LT complications, while additional factors, such as pre-transplant metabolic syndrome, may also predispose to post-LT DM. Additionally, glucocorticoids and immunosuppressive therapy play an important role in glycemic homeostasis after LT [78]. Nevertheless, the management of cirrhotic patients undergoing LT is complicated by the lack of data on the impact of long-term glycemic control and lack of specific guidelines regarding efficacy of anti-hyperglycemic agents in these individuals [79]. However, further prospective studies are needed in order to clarify the impact of new onset DM on survival and outcomes in LT recipients.

Conversely, post-cirrhotic DM (HD), which develops as a complication of LC and typically does not arise until LC has reached an advanced stage, seems to be potentially reversible (compared to T2DM) after LT, as few cases demonstrated [80]. As previously said, while IR is the primary event complicating LC, additional β-cell secretory impairment is crucial for the development of DM. LT, improving IR, may cure potential HD; nevertheless, the persistence of reduced β-cell function could contribute to ongoing insulin disturbance, making some patients eventually eligible for combined islet transplantation [81]. However, only prospective studies with precise temporal evaluation can differentiate new onset DM after LT from HD developing before LT and reversing subsequently, with further need for specific evaluation of the last-mentioned aspect.

Ultimately and most importantly, HD should therefore be distinguished from other types of DM and perceived as a well-defined clinical entity. However, the number of studies dealing with HD and the number of patients involved is relatively low; therefore, a firm recommendation cannot be issued. Although we presented the current definitions and outcomes of HD, its clinical implications may not be highly relevant in clinical practice and further evidence-based studies should be carried out in order to elucidate the impact of HD on LC evolution.

Moreover, the diagnosis of DM in patients with LC may not be easy, especially if taking into account post-cirrhotic DM (HD). Judging by the criteria used for the diagnosis of DM, we consider determining FPG and HbA1C alone as being insufficient for a complete evaluation of insulin homeostasis and glycemic status. Owing to the inherent drawback of a retrospective research, an accurate assessment of DM was lacking in most of the studies and many cases with potential DM may have been overlooked. More importantly, IR is predominant in patients with LC and may contribute per se to increased risk of decompensating events. However, IR was evaluated in only one study [22]. Therefore, we consider that the diagnosis of DM should be performed prospectively and that every patient with confirmed LC should undergo screening for DM, including an evaluation of IR and other GMDs.

Additionally, treatment of HD is challenging, given the risk of hypoglycemia and the high level of IR accompanied by hyperinsulinemia, leading to variable insulin requirement [12]. In addition, the impact of glucose control on clinical outcomes of patients with HD has not been evaluated [82].

Overall, this study provides an overview on the available evidence regarding the impact of DM on the risk of specific complications of LC. Additionally, it brings new perspectives on post-cirrhotic DM (HD) both as a complication of LC, as well as a risk factor for other decompensating events.

A limitation is represented by the paucity of clinical studies regarding specific complications of LC associating DM. More importantly, the cited studies were found to have a pronounced clinical and methodological heterogeneity, were predominantly retrospective, and blinding of the outcome assessors was not mentioned.

Nevertheless, there are some additional inherent limitations of a non-systematic approach. A systematic literature search could not be performed given that the study aimed to complete a summary of literature on a broad topic, covering the main complications and two additional outcomes of LC associating DM. High-quality evidence could not be provided due to heterogeneity of studies. A rigorous critical appraisal of included studies was not performed, but, however, the overall quality of evidence and potential risk of bias of some individual studies were subjectively reported in the Supplementary Materials and in the discussions above.

As future perspective, due to lack of good quality evidence regarding the impact of DM on renal function in patients with LC, further studies should be performed in this field.

In conclusion, patients with DM and LC are at higher risk for complications and may benefit from close monitoring of comorbidities during follow-up in order to reduce readmissions and mortality. However, prospective multicenter clinical trials are required to further validate these findings. Additionally, data on the impact of pre-cirrhotic versus post-cirrhotic DM (HD) on LC outcomes is insufficient and the clinical impact of HD on LC outcomes should be further evaluated.

## Figures and Tables

**Table 1:** The synthetic relationship between increased risk of hepatocellular carcinoma and presence of liver cirrhosis and/or diabetes mellitus.

Studies	Main Factors for Increased Risk of HCC								
	LC	DM	Increased Age	Gender M/*F	HBV	HCV	Thrombo-Cytopenia	High BMI	Steatosis
Wideroff L et al., 1997 [34]	-	+		+	-	-			
Tung HD et al., 2010 [39]		-		+	+	+	+	-	
Li Q et al., 2012 [36]	-	+		* +	+				
Zheng Z et al., 2013 [37]		+	+	+	+	+			
Karagozian et al., 2013 [42]	+	-							
Karagozian et al., 2013 (a subgroup analysis) [42]	-	+							
Orci LA et al., 2013 (recurrence of HCC after LT) [44]		+ (donor)						+ (donor)	+ (donor)
Liu TL et al., 2016 [10]	+	+	+						
Yang DM, 2016 [41]	+	-				+			
Yang DM, 2016 (pts. without HCV) [41]		+				-			
Shima T et al., 2019 [40]		+					+		
Yang DM et al., 2020 [43]	+	+	+						+

Legend: “+” = presence of association, “-“ = lack of association, HCC = hepatocellular carcinoma; LC = liver cirrhosis; DM = diabetes mellitus; M/F = male/female; HBV = hepatitis B virus; HCV = hepatitis C virus; BMI = body mass index; * + = risk for female higher than for male; LT = liver transplant; pts. = patients.

## Supplementary Materials

The following are available online at https://www.mdpi.com/2077-0383/10/2/262/s1, Table S1: Diabetes mellitus and hepatic encephalopathy, Table S2: Diabetes mellitus and variceal hemorrhage, Table S3: Diabetes mellitus and infectious complications of liver cirrhosis.

## References

[1] Garcia-Compean D., Jaquez-Quintana J.O., Gonzalez-Gonzalez J.A., Maldonado-Garza H. (2009). Liver Cirrhosis and Diabetes: Risk Factors, Pathophysiology, Clinical Implications and Management. World J. Gastroenterol..

[2] García-Compeán D., Jáquez-Quintana J.O., Lavalle-González F.J., Reyes-Cabello E., González-González J.A., Muñoz-Espinosa L.E., Vázquez-Elizondo G., Villarreal-Pérez J.Z., Maldonado-Garza H.J. (2012). The Prevalence and Clinical Characteristics of Glucose Metabolism Disorders in Patients with Liver Cirrhosis. A Prospective Study. Ann. Hepatol..

[3] Torbenson M., Chen Y.-Y., Brunt E., Cummings O.W., Gottfried M., Jakate S., Liu Y.-C., Yeh M.M., Ferrell L. (2006). Glycogenic Hepatopathy: An Underrecognized Hepatic Complication of Diabetes Mellitus. Am. J. Surg. Pathol..

[4] Hudacko R.M., Sciancalepore J.P., Fyfe B.S. (2009). Diabetic Microangiopathy in the Liver: An Autopsy Study of Incidence and Association with Other Diabetic Complications. Am. J. Clin. Pathol..

[5] Mohamed J., Nazratun Nafizah A.H., Zariyantey A.H., Budin S.B. (2016). Mechanisms of Diabetes-Induced Liver Damage: The Role of Oxidative Stress and Inflammation. Sultan Qaboos Univ. Med. J..

[6] Tilg H., Effenberger M. (2020). From NAFLD to MAFLD: When Pathophysiology Succeeds. Nat. Rev. Gastroenterol. Hepatol..

[7] Eslam M., Sanyal A.J., George J., Sanyal A., Neuschwander-Tetri B., Tiribelli C., Kleiner D.E., Brunt E., Bugianesi E., Yki-Järvinen H. (2020). MAFLD: A Consensus-Driven Proposed Nomenclature for Metabolic Associated Fatty Liver Disease. Gastroenterology.

[8] American Diabetes Association (2016). Erratum. Classification and Diagnosis of Diabetes. Sec. 2. In Standards of Medical Care in Diabetes-2016. Diabetes Care 2016; 39(Suppl. 1): S13–S22. Diabetes Care.

[9] Nishida T. (2017). Diagnosis and Clinical Implications of Diabetes in Liver Cirrhosis: A Focus on the Oral Glucose Tolerance Test. J. Endocr. Soc..

[10] Liu T.-L., Trogdon J., Weinberger M., Fried B., Barritt A.S. (2016). Diabetes Is Associated with Clinical Decompensation Events in Patients with Cirrhosis. Dig. Dis. Sci..

[11] Ahn S.B., Powell E.E., Russell A., Hartel G., Irvine K.M., Moser C., Valery P.C. (2020). Type 2 Diabetes: A Risk Factor for Hospital Readmissions and Mortality in Australian Patients with Cirrhosis. Hepatol. Commun..

[12] Kumar R. (2018). Hepatogenous Diabetes: An Underestimated Problem of Liver Cirrhosis. Indian J. Endocrinol. Metab..

[13] Holstein A., Hinze S., Thießen E., Plaschke A., Egberts E.-H. (2002). Clinical Implications of Hepatogenous Diabetes in Liver Cirrhosis. J. Gastroenterol. Hepatol..

[14] Elkrief L., Rautou P.-E., Sarin S., Valla D., Paradis V., Moreau R. (2016). Diabetes Mellitus in Patients with Cirrhosis: Clinical Implications and Management. Liver Int..

[15] Orsi E., Grancini V., Menini S., Aghemo A., Pugliese G. (2017). Hepatogenous Diabetes: Is It Time to Separate It from Type 2 Diabetes?. Liver Int..

[16] García-Compeán D., González-González J.A., Lavalle-González F.J., González-Moreno E.I., Villarreal-Pérez J.Z., Maldonado-Garza H.J. (2016). Hepatogenous Diabetes: Is It a Neglected Condition in Chronic Liver Disease?. World J. Gastroenterol..

[17] Sehrawat T., Jindal A., Kohli P., Thour A., Kaur J., Sachdev A., Gupta Y. (2018). Utility and Limitations of Glycated Hemoglobin (HbA1c) in Patients with Liver Cirrhosis as Compared with Oral Glucose Tolerance Test for Diagnosis of Diabetes. Diabetes Ther..

[18] Sigal S.H., Stanca C.M., Kontorinis N., Bodian C., Ryan E. (2006). Diabetes Mellitus Is Associated with Hepatic Encephalopathy in Patients with HCV Cirrhosis. Am. J. Gastroenterol..

[19] Butt Z., Jadoon N.A., Salaria O.N., Mushtaq K., Riaz I.B., Shahzad A., Hashmi A.M., Sarwar S. (2013). Diabetes Mellitus and Decompensated Cirrhosis: Risk of Hepatic Encephalopathy in Different Age Groups. J. Diabetes.

[20] Elkrief L., Chouinard P., Bendersky N., Hajage D., Larroque B., Babany G., Kutala B., Francoz C., Boyer N., Moreau R. (2014). Diabetes Mellitus Is an Independent Prognostic Factor for Major Liver-Related Outcomes in Patients with Cirrhosis and Chronic Hepatitis C. Hepatology.

[21] Jepsen P., Watson H., Andersen P.K., Vilstrup H. (2015). Diabetes as a Risk Factor for Hepatic Encephalopathy in Cirrhosis Patients. J. Hepatol..

[22] Yin X., Zhang F., Xiao J., Wang Y., He Q., Zhu H., Leng X., Zou X., Zhang M., Zhuge Y. (2019). Diabetes Mellitus Increases the Risk of Hepatic Encephalopathy after a Transjugular Intrahepatic Portosystemic Shunt in Cirrhotic Patients. Eur. J. Gastroenterol. Hepatol..

[23] Routhu M., Safka V., Routhu S.K., Fejfar T., Jirkovsky V., Krajina A., Cermakova E., Hosak L., Hulek P. (2017). Observational Cohort Study of Hepatic Encephalopathy after Transjugular Intrahepatic Portosystemic Shunt (TIPS). Ann. Hepatol..

[24] Labenz C., Nagel M., Kremer W.M., Hilscher M., Schilling C.A., Toenges G., Kuchen R., Schattenberg J.M., Galle P.R., Wörns M.-A. (2020). Association between Diabetes Mellitus and Hepatic Encephalopathy in Patients with Cirrhosis. Aliment. Pharmacol. Ther..

[25] Jeon H.K., Kim M.Y., Baik S.K., Park H.J., Choi H., Park S.Y., Kim B.R., Hong J.H., Jo K.W., Shin S.Y. (2013). Hepatogenous Diabetes in Cirrhosis Is Related to Portal Pressure and Variceal Hemorrhage. Dig. Dis. Sci..

[26] Yang C.-H., Chiu Y.-C., Chen C.-H., Chen C.-H., Tsai M.-C., Chuah S.-K., Lee C.-H., Hu T.-H., Hung C.-H. (2014). Diabetes Mellitus Is Associated with Gastroesophageal Variceal Bleeding in Cirrhotic Patients. Kaohsiung J. Med. Sci..

[27] Cammà C., Petta S., Di Marco V., Bronte F., Ciminnisi S., Licata G., Peralta S., Simone F., Marchesini G., Craxì A. (2009). Insulin Resistance Is a Risk Factor for Esophageal Varices in Hepatitis C Virus Cirrhosis. Hepatology.

[28] Khafaga S., Khalil K., Mohamed A., Miada M., Mahmoud S., Mohammad M. (2015). Acute Variceal Bleeding in Patients with Liver Cirrhosis with and without Diabetes. Liver Res. Open J..

[29] Tergast T.L., Laser H., Gerbel S., Manns M.P., Cornberg M., Maasoumy B. (2018). Association between Type 2 Diabetes Mellitus, HbA1c and the Risk for Spontaneous Bacterial Peritonitis in Patients with Decompensated Liver Cirrhosis and Ascites. Clin. Transl. Gastroenterol..

[30] Ko M.-C., Lin W.-H., Martini S., Chang Y.-H., Chiu C.-T., Li C.-Y. (2019). A Cohort Study of Age and Sex Specific Risk of Pyogenic Liver Abscess Incidence in Patients with Type 2 Diabetes Mellitus. Medicine.

[31] Bossen L., Dam G.A., Vilstrup H., Watson H., Jepsen P. (2019). Diabetes Does Not Increase Infection Risk or Mortality Following an Infection in Patients with Cirrhosis and Ascites. JHEP Rep..

[32] Ramachandran T.M., Rajneesh A.H.R., Zacharia G.S., Adarsh R.P. (2017). Cirrhosis of Liver and Diabetes Mellitus: The Diabolic Duo?. J. Clin. Diagn. Res..

[33] Rosenblatt R., Atteberry P., Tafesh Z., Ravikumar A., Crawford C.V., Lucero C., Jesudian A.B., Brown R.S., Kumar S., Fortune B.E. (2020). Uncontrolled Diabetes Mellitus Increases Risk of Infection in Patients with Advanced Cirrhosis. Dig. Liver Dis..

[34] Wideroff L., Gridley G., Chow W.-H., Linet M., Mellemkjaer L., Olsen J.H., Keehn S., Borch-Johnsen K. (1997). Cancer Incidence in a Population-Based Cohort of Patients Hospitalized with Diabetes Mellitus in Denmark. J. Natl. Cancer Inst..

[35] El-Serag H.B., Hampel H., Javadi F. (2006). The Association between Diabetes and Hepatocellular Carcinoma: A Systematic Review of Epidemiologic Evidence. Clin. Gastroenterol. Hepatol..

[36] Li Q., Li W.-W., Yang X., Fan W.-B., Yu J.-H., Xie S.-S., Liu L., Ma L.-X., Chen S.-J., Kato N. (2012). Type 2 Diabetes and Hepatocellular Carcinoma: A Case-Control Study in Patients with Chronic Hepatitis B. Int. J. Cancer.

[37] Zheng Z., Zhang C., Yan J., Ruan Y., Zhao X., San X., Mao Y., Sun Q., Zhang K., Fan Z. (2013). Diabetes Mellitus Is Associated with Hepatocellular Carcinoma: A Retrospective Case-Control Study in Hepatitis Endemic Area. PLoS ONE.

[38] Chao L.-T., Wu C.-F., Sung F.-Y., Lin C.-L., Liu C.-J., Huang C.-J., Tsai K.-S., Yu M.-W. (2011). Insulin, Glucose and Hepatocellular Carcinoma Risk in Male Hepatitis B Carriers: Results from 17-Year Follow-up of a Population-Based Cohort. Carcinogenesis.

[39] Tung H.-D., Wang J.-H., Tseng P.-L., Hung C.-H., Kee K.-M., Chen C.-H., Chang K.-C., Lee C.-M., Changchien C.-S., Chen Y.-D. (2010). Neither Diabetes Mellitus nor Overweight Is a Risk Factor for Hepatocellular Carcinoma in a Dual HBV and HCV Endemic Area: Community Cross-Sectional and Case–Control Studies. Am. J. Gastroenterol..

[40] Shima T., Uto H., Ueki K., Kohgo Y., Yasui K., Nakamura N., Nakatou T., Takamura T., Kawata S., Notsumata K. (2019). Hepatocellular Carcinoma as a Leading Cause of Cancer-Related Deaths in Japanese Type 2 Diabetes Mellitus Patients. J. Gastroenterol..

[41] Yang J.D., Mohamed H.A., Cvinar J.L., Gores G.J., Roberts L.R., Kim R.W. (2016). Diabetes Mellitus Heightens the Risk of Hepatocellular Carcinoma Except in Patients with Hepatitis C Cirrhosis. Am. J. Gastroenterol..

[42] Karagozian R., Baker E., Houranieh A., Leavitt D., Baffy G. (2013). Risk Profile of Hepatocellular Carcinoma Reveals Dichotomy among US Veterans. J. Gastrointest. Cancer.

[43] Yang J.D., Ahmed F., Mara K.C., Addissie B.D., Allen A.M., Gores G.J., Roberts L.R. (2020). Diabetes Is Associated with Increased Risk of Hepatocellular Carcinoma in Patients with Cirrhosis from Nonalcoholic Fatty Liver Disease. Hepatology.

[44] Orci L.A., Berney T., Majno P.E., Lacotte S., Oldani G., Morel P., Mentha G., Toso C. (2015). Donor Characteristics and Risk of Hepatocellular Carcinoma Recurrence after Liver Transplantation. Br. J. Surg..

[45] Aleksandrova K., Boeing H., Nöthlings U., Jenab M., Fedirko V., Kaaks R., Lukanova A., Trichopoulou A., Trichopoulos D., Boffetta P. (2014). Inflammatory and Metabolic Biomarkers and Risk of Liver and Biliary Tract Cancer. Hepatology.

[46] Schiaffini R., Liccardo D., Alisi A., Benevento D., Cappa M., Cianfarani S., Nobili V. (2016). Early Glucose Derangement Detected by Continuous Glucose Monitoring and Progression of Liver Fibrosis in Nonalcoholic Fatty Liver Disease: An Independent Predictive Factor?. Horm. Res. Paediatr..

[47] Golabi P., Bush H., Stepanova M., Locklear C.T., Jacobson I.M., Mishra A., Trimble G., Erario M., Venkatesan C., Younossi I. (2018). Liver Transplantation (LT) for Cryptogenic Cirrhosis (CC) and Nonalcoholic Steatohepatitis (NASH) Cirrhosis: Data from the Scientific Registry of Transplant Recipients (SRTR): 1994 to 2016. Medicine.

[48] Liu F.-C., Lin J.-R., Chen H.-P., Tsai Y.-F., Yu H.-P. (2016). Prevalence, Predictive Factors, and Survival Outcome of New-Onset Diabetes after Liver Transplantation: A Population-Based Cohort Study. Medicine.

[49] Lieber S.R., Lee R., Jiang Y., Reuter C., Watkins R., Szempruch K., Gerber D.A., Desai C.S., DeCherney G.S., Barritt A.S. (2019). The Impact of Post-Transplant Diabetes Mellitus on Liver Transplant Outcomes. Clin. Transplant..

[50] Aravinthan A.D., Fateen W., Doyle A.C., Venkatachalapathy S.V., Issachar A., Galvin Z., Sapisochin G., Cattral M.S., Ghanekar A., McGilvray I.D. (2019). The Impact of Preexisting and Post-Transplant Diabetes Mellitus on Outcomes Following Liver Transplantation. Transplantation.

[51] Kawaguchi T., Taniguchi E., Itou M., Sakata M., Sumie S., Sata M. (2011). Insulin Resistance and Chronic Liver Disease. World J. Hepatol..

[52] Chow L.S., Albright R.C., Bigelow M.L., Toffolo G., Cobelli C., Nair K.S. (2006). Mechanism of Insulin’s Anabolic Effect on Muscle: Measurements of Muscle Protein Synthesis and Breakdown Using Aminoacyl-TRNA and Other Surrogate Measures. Am. J. Physiol. Endocrinol. Metab..

[53] Sepehrinezhad A., Zarifkar A., Namvar G., Shahbazi A., Williams R. (2020). Astrocyte Swelling in Hepatic Encephalopathy: Molecular Perspective of Cytotoxic Edema. Metab. Brain. Dis..

[54] Matulewicz N., Karczewska-Kupczewska M. (2016). Insulin Resistance and Chronic Inflammation. Postepy Higieny i Medycyny Doswiadczalnej.

[55] Handra C., Coman O.A., Coman L., Enache T., Stoleru S., Sorescu A.-M., Ghita I., Fulga I. (2019). The Connection between Different Neurotransmitters Involved in Cognitive Processes. FARMACIA.

[56] Basu S., Zethelius B., Helmersson J., Berne C., Larsson A., Arnlöv J. (2011). Cytokine-Mediated Inflammation Is Independently Associated with Insulin Sensitivity Measured by the Euglycemic Insulin Clamp in a Community-Based Cohort of Elderly Men. Int. J. Clin. Exp. Med..

[57] Jun D.W., Kim K.T., Lee O.Y., Chae J.D., Son B.K., Kim S.H., Jo Y.J., Park Y.S. (2010). Association between Small Intestinal Bacterial Overgrowth and Peripheral Bacterial DNA in Cirrhotic Patients. Dig. Dis. Sci..

[58] Elwir S., Rahimi R.S. (2017). Hepatic Encephalopathy: An Update on the Pathophysiology and Therapeutic Options. J. Clin. Transl. Hepatol..

[59] Takahashi T., Yoshihisa A., Sugimoto K., Yokokawa T., Misaka T., Kaneshiro T., Oikawa M., Kobayashi A., Nakazato K., Ishida T. (2018). Associations between Diabetes Mellitus and Pulmonary Hypertension in Chronic Respiratory Disease Patients. PLoS ONE.

[60] Pugliese D., Lee S.S., Koshy A., Cerini R., Ozier Y., Lebrec D. (1988). Systemic and Splanchnic Hemodynamic Effects of Intravenous Hypertonic Glucose in Patients with Cirrhosis. Hepatology.

[61] Majid S., Azam Z., Shah H.A., Salih M., Hamid S., Abid S., Jafri W. (2009). Factors Determining the Clinical Outcome of Acute Variceal Bleed in Cirrhotic Patients. Indian J. Gastroenterol..

[62] Eslam M., Ampuero J., Jover M., Abd-Elhalim H., Rincon D., Shatat M., Camacho I., Kamal A., Lo Iacono O., Nasr Z. (2013). Predicting Portal Hypertension and Variceal Bleeding Using Non-Invasive Measurements of Metabolic Variables. Ann. Hepatol..

[63] Maruyama H., Kobayashi K., Kiyono S., Yokosuka O. (2017). Interrelationship between Insulin Resistance and Portal Haemodynamic Abnormality in Cirrhosis. Int. J. Med. Sci..

[64] Wasfy E., Elkassas G., Elnawasany S., Elkasrawy K., Abd-Elsalam S., Soliman S., Badawi R. (2018). Predicting Esophageal Varices in Cirrhotic Hepatitis C Virus Patients Using Noninvasive Measurement of Insulin Resistance Variables. Endocr. Metab. Imunne Disord. Drug Targets.

[65] Djiambou-Nganjeu H. (2019). Relationship between Portal HTN and Cirrhosis as a Cause for Diabetes. J. Transl. Int. Med..

[66] Cheruvattath R., Balan V. (2007). Infections in Patients with End-Stage Liver Disease. J. Clin. Gastroenterol..

[67] Irvine K.M., Ratnasekera I., Powell E.E., Hume D.A. (2019). Causes and Consequences of Innate Immune Dysfunction in Cirrhosis. Front. Immunol..

[68] Gupta S., Koirala J., Khardori R., Khardori N. (2007). Infections in Diabetes Mellitus and Hyperglycemia. Infect. Dis. Clin. North Am..

[69] Tsujimoto T., Kajio H., Sugiyama T. (2017). Association between Hyperinsulinemia and Increased Risk of Cancer Death in Nonobese and Obese People: A Population-Based Observational Study: Cancer Mortality and Hyperinsulinemia without Obesity. Int. J. Cancer.

[70] Cannata D., Fierz Y., Vijayakumar A., LeRoith D. (2010). Type 2 Diabetes and Cancer: What Is the Connection? Special Feature-Type 2 Diabetes and Cancer. Mt. Sinai J. Med..

[71] Singh M.K., Das B.K., Choudhary S., Gupta D., Patil U.K. (2018). Diabetes and Hepatocellular Carcinoma: A Pathophysiological Link and Pharmacological Management. Biomed. Pharmacother..

[72] Imai K., Takai K., Hanai T., Suetsugu A., Shiraki M., Shimizu M. (2019). Homeostatic Model Assessment of Insulin Resistance for Predicting the Recurrence of Hepatocellular Carcinoma after Curative Treatment. Int. J. Mol. Sci..

[73] Estes C., Razavi H., Loomba R., Younossi Z., Sanyal A.J. (2018). Modeling the Epidemic of Nonalcoholic Fatty Liver Disease Demonstrates an Exponential Increase in Burden of Disease: Estes et al. Hepatology.

[74] Dhamija E., Paul S., Kedia S. (2019). Non-Alcoholic Fatty Liver Disease Associated with Hepatocellular Carcinoma: An Increasing Concern. Indian J. Med. Res..

[75] European Association for the Study of the Liver (2018). European Association for the Study of the Liver EASL Clinical Practice Guidelines: Management of Hepatocellular Carcinoma. J. Hepatol..

[76] Pennisi G., Celsa C., Giammanco A., Spatola F., Petta S. (2019). The Burden of Hepatocellular Carcinoma in Non-Alcoholic Fatty Liver Disease: Screening Issue and Future Perspectives. Int. J. Mol. Sci..

[77] Bhat V., Tazari M., Watt K.D., Bhat M. (2018). New-Onset Diabetes and Preexisting Diabetes Are Associated with Comparable Reduction in Long-Term Survival After Liver Transplant: A Machine Learning Approach. Mayo Clin. Proc..

[78] Peláez-Jaramillo M.J., Cárdenas-Mojica A.A., Gaete P.V., Mendivil C.O. (2018). Post-Liver Transplantation Diabetes Mellitus: A Review of Relevance and Approach to Treatment. Diabetes Ther..

[79] Grancini V., Resi V., Palmieri E., Pugliese G., Orsi E. (2019). Management of Diabetes Mellitus in Patients Undergoing Liver Transplantation. Pharmacol. Res..

[80] Pallayova M., Wilson V., John R., Taheri S. (2013). Liver Transplantation: A Potential Cure for Hepatogenous Diabetes?. Diabetes Care.

[81] Perseghin G., Mazzaferro V., Sereni L.P., Regalia E., Benedini S., Bazzigaluppi E., Pulvirenti A., Leão A.A.S., Calori G., Romito R. (2000). Contribution of Reduced Insulin Sensitivity and Secretion to the Pathogenesis of Hepatogenous Diabetes: Effect of Liver Transplantation. Hepatology.

[82] Hamed A.E., Elwan N., Naguib M., Elwakil R., Esmat G., El Kassas M., Abd-Elsalam S., Moussa S. (2019). Diabetes Association with Liver Diseases: An Overview for Clinicians. Endocr. Metab. Immune Disord. Drug Targets.

