# Supplementary material for: Association between Liver Cirrhosis and Diabetes Mellitus: A Review on Hepatic Outcomes
Source: J Clin Med. 2021 Jan 12;10(2):262. doi: 10.3390/jcm10020262 (PMC7827383; doi:10.3390/jcm10020262)
Supplement: Supplementary file 1 [file jcm-10-00262-s001.zip › Table S3 (1).docx]

**Table S3: Diabetes mellitus and infectious complications of liver cirrhosis.**

| **First author, year, country of first author, reference** | **Population and Selection** | **Aim and outcome assessment** | **Study design** | **Comparison** | | **Exclusion criteria** | **Main outcomes** | **Bias/Limitations** |
| --- | --- | --- | --- | --- | --- | --- | --- | --- |
|  |  |  |  | **DM** | **Non-DM** |  |  |  |
| Tergast T.L.  2018  Germany  [1] | - 600 consecutive pts. with decompensated LC  - 475 pts. included in final sample  - 279 M, 196 F  - mean age 55.34 y  - median MELD = 19.45  - T2DM was diagnosed based on history | - To assess the impact of T2DM and HbA1c values on the incidence of SBP in pts. with LC and ascites  - SBP was diagnosed based on a polymorphouclear leucocyte count ≥250 cells/mm³ or a total nucleus containing cell count ≥500 cells/mm³ in ascitic fluid  - Median follow-up was 266 days  - All pts. had screening paracentesis at inclusion | Prospective, case-control, longitudinal, January 2012 - June 2016 | - 118 (25%)  - mean age 58 y  - median MELD = 18.97 | - 357 (75%)  - mean age 54.44 y  - median MELD = 19.61 | - Secondary intraabdominal infection, HIV or congenital immune dysfunction  - History of stem cell or solid organ transplant (except LT)  - Evidence of malignancy (except HCC within the Milan criteria)  - Pts. with SBP at screening paracentesis were excluded (only pts. with SBP at first paracentesis (n=151), as well as those with history of SBP (n=70) were considered) | - T2DM pts. had an increased risk for developing SBP during follow-up (HR: 1.51, p=0.03)  - SBP incidence was significantly higher in T2DM pts. with HbA1c values ≥6.4% than in those with HbA1c values <6.4% (HR: 4.21, p=0.0002)  - T2DM pts. with HbA1c <6.4% at baseline had a similar risk for SBP as those without T2DM (HR: 0.93, p=0.78) | - No clear definition of diagnostic criteria for T2DM  - IR was not evaluated  - HbA1c values were available for only 86% of pts. and there was no access to multiple HbA1c values over the follow-up period |
| Ko M.C.  2019  Taiwan  [2] | - Population from National Health Insurance Research Database: 613,921 T2DM pts. and 614,613 age- and sex-matched controls  - mean age 60 y  - Diagnosis of T2DM was based on ICD-9-CM | - To investigate the age-sex-specific incidence and relative risk of PLA in pts. with T2DM and to assess the joint effects of T2DM and other clinical risk factors for PLA on PLA incidence  - Median follow-up was 11 years  - Diagnosis of PLA was based on ICD-9-CM | Retrospective, case-control, database study, January 2000 – 31 December 2010 | - 7.58% of T2DM pts. had LC | - 4.03% of controls had LC | - Controls with prior diagnostic of PLA (from Jan 1 1997 to index date: Jan 1, 2000) | - T2DM was significantly associated with increased risk of PLA (HR, 2.88; 95% CI: 2.73–3.04), especially in male and younger subjects (<45 y)  - Biliary tract diseases and LC added additional risk for PLA in pts. with T2DM, with a HR of 8.60 (95% CI: 7.87–9.40) and 7.52 (95% CI: 6.58–8.59), respectively | - Misclassification bias due to reliance on ICD codes (possible undiagnosed T2DM cases in controls)  - Possible exclusion of some eligible pts.  - T2DM pts. had more LC, which is a risk factor for PLA |
| Bossen L.  2019  Denmark  [3] | - 1198 pts. with LC and ascites, included in 3 concomitant satavaptan trials  - The study had different target populations:  I. Pts. with diuretic-manageable ascites (n=462),  II. Pts. with diuretic-manageable ascites and occasional therapeutic paracentesis (n=496),  III. Pts. with diuretic-resistant ascites managed primarily with therapeutic paracentesis (n=240)  - Severity of hepatic dysfunction was assessed through Child-Pugh and MELD scores | - To compare the risk of infections and mortality following an infection in pts. with LC, with or without DM with an *a priori* expectation that DM would increase this risk  - Pts. were treated with satavaptan for 1 year and were followed until infection, death or to the date of the final drug safety assessment  - Similar proportions of DM and non-DM pts. (13% vs. 12%) took a quinolone antibiotic | Database study; data from three multinational RCTs (second and third stopped early due to poor benefit-risk ratio), July 2006 - December 2008 | - 289 (24%).  - 73% M  - median MELD = 14 | - 909 (76%)  - 69% M  - median MELD = 15 | - SBP or VH in the 10 days before randomisation; HE ≥ grade 2 at randomisation; functioning TIPS; serum creatinine >150 μmol/L, serum potassium >5.0 mmol/L, serum sodium >143 mmol/L, serum bilirubin >150 μmol/L, INR>3.0, platelets <30,000/mm3, neutrophils <1,000/mm3; systolic blood pressure <80 mmHg or symptomatic orthostatic hypotension;  HCC exceeding Milan criteria; use of a potent modifier of cytochrome P450 3A pathway and drugs that increase the risk of QT interval prolongation | - 18% of diabetics vs. 14% of non-diabetics had previous SBP  - DM did not increase the HR of infections (aHR 1.08; 95% CI: 0.87-1.35) or the mortality following an infection (aHR 0.93; 95% CI: 0.64-1.35)  - The 1-year cumulative  risk of infection was 44.5% (95% CI: 41.2–47.8); satavaptan use did not influence  the rate of infections (aHR 0.98; 95% CI: 0.81–1.19) | - The study protocol did not give explicit criteria for the diagnosis of DM |

**Legend:** pts. = patients; LC = liver cirrhosis; M =male; F = female MELD = Model for End-stage Liver Disease score; T2DM = type 2 diabetes mellitus; SBP = spontaneous bacterial peritonitis; HbA1c = glycosylated hemoglobin; LT = liver transplant; HCC = hepatocellular carcinoma; NASH = non-alcoholic steatohepatitis; PLA = pyogenic liver abscess; ICD-9-CM = International Classification of Diseases, ninth revision, Clinical Modification; PPI = proton pump inhibitors; VH = variceal hemorrhage; HE = hepatic encephalopathy; TIPS = Transjugular Intrahepatic Portosystemic Shunt

**References**

1. Tergast, T.L.; Laser, H.; Gerbel, S.; Manns, M.P.; Cornberg, M.; Maasoumy, B. Association Between Type 2 Diabetes Mellitus, HbA1c and the Risk for Spontaneous Bacterial Peritonitis in Patients with Decompensated Liver Cirrhosis and Ascites. *Clin Transl Gastroenterol* **2018**, *9*, 189, doi:10.1038/s41424-018-0053-0.

2. Ko, M.-C.; Lin, W.-H.; Martini, S.; Chang, Y.-H.; Chiu, C.-T.; Li, C.-Y. A Cohort Study of Age and Sex Specific Risk of Pyogenic Liver Abscess Incidence in Patients with Type 2 Diabetes Mellitus. *Medicine (Baltimore)* **2019**, *98*, e15366, doi:10.1097/MD.0000000000015366.

3. Bossen, L.; Dam, G.A.; Vilstrup, H.; Watson, H.; Jepsen, P. Diabetes Does Not Increase Infection Risk or Mortality Following an Infection in Patients with Cirrhosis and Ascites. *JHEP Rep* **2019**, *1*, 265–269, doi:10.1016/j.jhepr.2019.07.008.
