# Supplementary material for: Association between Liver Cirrhosis and Diabetes Mellitus: A Review on Hepatic Outcomes
Source: J Clin Med. 2021 Jan 12;10(2):262. doi: 10.3390/jcm10020262 (PMC7827383; doi:10.3390/jcm10020262)
Supplement: Supplementary file 1 [file jcm-10-00262-s001.zip › Table S1 (1).docx]

Table S1. Diabetes mellitus and hepatic encephalopathy

| **First author, year, country of first author, reference** | **Population and Selection** | **Aim and Outcome assessment** | **Study design** | **Comparison** | | **Exclusion criteria** | **Main outcomes** | **Bias/Limitations** |
| --- | --- | --- | --- | --- | --- | --- | --- | --- |
|  |  |  |  | **DM** | **Non-DM** |  |  |  |
| Sigal S.H.  2006  USA  [1] | - 65 pts. with HCV-related LC attending LT  -50 M, 15 F  - mean age 52 y (range 20–75 y)  - median MELD =15 (range 6-32)  - median modified CP score * =7 (range 4-12)  - DM was diagnosed based on history and elevated FPG (>126 mg/dl) | - To assess if DM would predispose to or exacerbate the severity of HE  - Frequency and severity of HE were assessed clinically (West-Haven criteria 1-4; insomnia, day/night reversal, poor concentration, irritability, asterixis, constructional apraxia) and by neuropsychometric testing (Trail Making Tests Part A and B, WAIS-III Digit Symbol Test)  - HE was categorized as *mild* (minimal and persistent stage 1) and *severe* (persistent stage 2 or episodic stage 3 or 4) | Prospective, cross-sectional | - 20 (31%)  - modified CP score* A/B/C = 5/12/3 | - 45 (69%)  - modified CP score* A/B/C = 8/17/20 | - Recent GI bleeding, active infection, creatinine >2.0 mg/dl, active alcohol consumption, history of TIPS, use of benzodiazepines, methadone, narcotics, agents that alter GI motility, except lactulose | - HE was diagnosed in 19 (95%) pts. with DM (35% mild; 60% severe) and 35 (78%) pts. without DM (58% mild; 20% severe)  - DM was a significant independent risk factor for HE (p=0.0008)  - When stratified by modified CP score*, DM pts. were  more likely to have severe HE (A vs. C, p=0.002; B vs. C, p=0.007) | - Small sample of pts.; gender heterogeneity  - Study duration not mentioned  - IR was not evaluated  - Modified CP score* not previously validated  - Definitions of mild/severe HE not standardized |
| Butt Z.  2013  Pakistan  [2] | - 352 consecutive pts. with decompensated LC (71.6% HCV-related, 12.2% HBV-related)  - 211 M, 141 F  - 33.5% had > 60y  - 25.7 % had MELD <15  - modified CP score* was used (A/B/C = 10.9%/42.3%/46.8%)  - DM was diagnosed based on elevated FPG (>126 mg/dl) on 2 consecutive days and HbA1c ≥6.5% - only T2DM pts. were included | - To determine the relationship between DM, age and presence and severity of HE  - All pts. were examined for HE at admission according to West-Haven criteria | Prospective, cross-sectional, multicenter (2 major hospitals), June 2010 - Dec 2011 | - 118 (33.5%)  - mean age 54 y  - 57.6% M  - modified CP score* A/B/C = 7.9%/43.8%/48.3%  - MELD ≥15 in 76.2%  - HE grade 4: 12.7% | - 234 (66.5%)  - mean age 50.8 y  - 61.1% M  - modified CP score* A/B/C = 12.5%/41.5%/46%  - MELD ≥ 15 in 73.2%  - HE grade 4: 5.6% | - Not mentioned | - HE at admission: 58.5% diabetics, vs. 42.6% non-diabetics (p=0.03)  - Severity of HE was greater in diabetics (p trend=0.01)  - DM, older age (>60 y) and male gender were independently associated with HE (p=0.03, 0.01 and 0.02, respectively) | - IR was not evaluated  - Modified CP score* not previously validated  - No exclusion criteria before HE evaluation**  - HE evaluated only at admission |
| Elkrief L.  2014  Canada  [3] | - 1380 pts. with chronic HCV infection; 348 with LC  - 236 M, 112 F  - mean age 59 y  - median MELD = 10 (range 8-14)  - DM was diagnosed based on ICD10 codes and retrospective analysis of medical records | - To evaluate whether gender, age, DM, alcohol abuse and HIV-HBV co-infection could influence liver-related outcomes, including HE  - Pts. were followed-up every 6 months; median follow-up = 55 months  - HE was diagnosed based on ICD10 codes and retrospective analysis of medical records | Retrospective, cohort, single-centered, January 1 2006 – March 2013 | - 139 (40%)  - MELD < 10 in 40% | - 209 (40%) | - LT before January 1 2006 (n=53)  - Pts. receiving LT were excluded from follow-up  - Among LC pts. 178 died (49%); 33 were lost on follow-up (9%) | - 31 (9%) had HE at inclusion; 36  (11%) developed HE during follow-up  - DM was associated with HE at inclusion (p<0.001)  - DM only clearly influenced LT-free  survival in pts. with LC and MELD <10 at baseline (p<0.005) | - Possible omission of undiagnosed DM and HE due to retrospective analysis  - HE severity assessment not performed  - No exclusion criteria before HE evaluation** |
| Jepsen P.  2015  Denmark  [4] | - 1 198 pts. with LC and ascites, included in 3 concomitant satavaptan RCTs  - 862 pts. in final sample | - To examine if DM increases the risk of first-time overt HE  - Pts. were examined every 4 weeks during 1 y follow-up  - Frequency and severity of HE were assessed clinically (West-Haven 2,3,4); first-time overt HE episodes and likely precipitating factors were recorded  - FBG [hypoglycemia (<70 mg/dl), euglycemia (70 to 179 mg/dl), or hyperglycemia (>180 mg/dl)] was examined as a risk factor for HE development in DM pts. | Database study; data from three multinational RCTs (second and third stopped early due to poor benefit-risk ratio), July 2006 - December 2008 | - 193 (22%)  - mean age 61 y  - 74% M  - NASH-cirrhosis: 18%  - median MELD = 13.9  - CP class A/B/C = 13%/74%/13% | - 669 (78%)  - mean age 56 y  - 67% M  - NASH-cirrhosis: 5%  - median MELD = 16.3  - CP class A/B/C = 9%/68%/23% | - Previous overt HE (n=306) or overt HE at randomization (n=26)  - Lack of recorded CP class (n=3) or serum biochemistry (n=1)  - functioning TIPS  - VB or SBP  in the last 10 days before randomization - serum creatinine >151 μmol/L, serum potassium >5 mmol/L, serum sodium >143  mmol/L, serum bilirubin >150 μmol/L, INR >3.0, platelets <30*109/L, neutrophils <1*109/L  - Malignancy - all pts. with cancer were excluded at randomization (including HCC exceeding Milan criteria) | - 1-year risk of first-time overt HE was 18.1% overall: 26% for DM vs. 15.8% for non-DM pts.  - Diabetics had more severe first-time episodes of overt HE: 64% progressed beyond grade 2 (39% grade 3, 25% grade 4) compared to 42% among non-diabetics (15% grade 3, 27% grade 4) (p=0.01)  - There was no difference in precipitating factors btw. DM and non-DM pts. (p=0.84)  - The rate of first-time overt HE was not higher in hyperglycemic pts. (aHR for hyperglycemia vs. euglycemia = 1.14, 95% CI: 0.49 to 2.64) | - No explicit criteria for the diagnosis of DM in the study protocol  - IR and HbA1c levels were not evaluated  - minimal HE was not evaluated (although previous overt HE was excluded, minimal HE before randomization could have been missed)  - Impact of satavaptan on HE or precipitating factors of HE not previously assessed for diabetic pts. |
| Liu T-L.  2016  USA  [5] | - 307 050 working-age, insured pts. with compensated LC  - 72 731 pts. in final sample  - 39 065 M, 33 666 F  - 8.5% < 40 y, 21.5% > 65 y  - Decompensating events = ascites, SBP, VH, HE, acute renal failure, HCC  - Diagnosis of DM and decompensating events based on ICD-9-CM codes  - DM severity was assessed based on DM complications and DM medication | - To examine the risk of decompensation in pts. with compensated LC and DM  - HE diagnosed based on ICD-9-CM codes | Retrospective, cohort; data from MarketScan Commercial Claims and Encounters and Medicare Supplemental Databases, 1 January 2000 – 31 December 2013 | - 20 477 (28.15%)  - 3.3% < 40 y  - 27.69% > 65 y | - 52 234 (71.85%)  - 10.54% < 40 y  - 19.16% > 65 y | - Age > 18 y  - Pts. with previous treatment for HE (lactulose, rifaximin)  - Diagnosis of DM before first diagnosis of LC  - Pts. with LC only who had DM medication  - Enrollment < 6 months before and after the first diagnosis of LC (n=205 437)  - Pts. with decompensation events or who dropped out of the system prior to the first diagnosis of compensated LC  - Pts. with HIV infection  - Pts. with LT | - Pts. with DM had more decompensating events (33.3% vs. 25.9%, p<0.01) and higher odds of developing any decompensation (OR=1.14; 95% CI: 1.08-1.21)  - HE was more prevalent in diabetics 15.35% vs. 13.26% (p<0.01) | - No exclusion criteria before HE evaluation**  - Due to retrospective analysis, undiagnosed DM or decompensated LC could have been missed  - Laboratory data were not available  - Population of the study was selected from working-age, insured pts.; uninsured, vulnerable pts. may have higher prevalence of DM or decompensated LC |
| Routhu M,  2017  UK  [6] | - 895 pts. with LC  - 678 pts. in final sample  - 433 M, 245 F  - mean age 54.5 y  - 55% had alcoholic cirrhosis  - CP A/B/C = 27%/47%/26% | - To study the association of preexisting  patient characteristics with the development of post-TIPS HE  - Follow-up at 1, 3 and 6 months after TIPS and every 3-6 months  thereafter | Retrospective, cohort, single-centered, September 1992 – August 2011 | - 192 (28%) | - 486 (72%) | - Age < 18 y  - Unsuccessful TIPS  - Pts. with pre-TIPS HE | - DM was a strong predictor of HE  - Diabetics or oral hypoglycemic agents had an OR of 1.487 (95% CI: 0.923 - 2.397) for developing HE  - Diabetics on insulin had an OR of 1.863 (95% CI: 1.070 - 3.244) of developing HE | - Diagnostic methods for DM and HE not clearly mentioned  - No exclusion criteria before HE evaluation**  - 125 pts. were lost during follow-up |
| Yin X.  2019  China  [7] | - 436 pts. with LC who received TIPS for VH, refractory ascites, or portal vein thrombosis (3.4%, 85.6%, and 2.1%, respectively).  - 278 M, 158 F  - mean age 55.3 ± 11.8 y  - CP class A/B/C = 32.8%/62.2%/3.0%  - DM was diagnosed based on FPG (≥126 mg/dl), symptoms of hyperglycemia and RPG (≥200 mg/dl) | - To examine the effect of DM on the rate of overt HE after TIPS  - Overt HE was diagnosed based on mental abnormalities (personality and behavior changes), neurological abnormalities (coma), disorientation and asterixis (as marker symptoms) and graded based on West-Haven criteria (2, 3 or 4)  - Pts. were examined monthly for the first 3 months after TIPS, then every 1–3 months, or if needed  - Median follow-up was 40.5 months | Retrospective, cohort, single-centered, 2008 - 2016 | - 85 (19.5%)  - Mean age 62 y  - alcoholic cirrhosis: 15.3%  - CP score A/B/C = 27%/72%/1%  - median MELD = 9.1 | - 351 (80.5%)  - Mean age 53.7 y  - alcoholic cirrhosis: 8.5%  - CP score A/B/C = 36%/60%/4%  - median MELD = 9.2 | - Unsuccessful TIPS (n=1)  - Overt HE before TIPS (n=12) | - 1-year risk of first-time overt HE was 26.6% overall: 40.0% for diabetics and 23.4% for non-diabetics  - Pts. with DM had a higher overall prevalence of HE (44.7% vs. 25.1%, p<0.001) and higher odds for HE (OR=1.901; 95% CI: 1.131–3.195)  - Older pts. had higher odds for overt HE after TIPS (OR=1.031; 95% CI: 1.101–1.053) | - IR was not evaluated  - changes in blood sugar after TIPS could affect HE  - No psychometric testing for minimal HE – minimal HE before TIPS was not evaluated  - No definition for old age  - No exclusion criteria before HE evaluation**  - VB before TIPS was not correlated with HE – could affect HE risk especially in diabetics |
| Labenz C.  2020  Germany  [8] | - 240 pts. with LC of any etiology (mostly alcohol-related, 33.8%)  - 137 M, 103 F  - mean age 60y  - median MELD = 10 (range 8-14)  - DM was diagnosed based on HbA1c values ≥6.5% (measured for all pts. at inclusion) | - To investigate a potential association btw. DM/glycemic control and covert HE or development of overt HE  - Overt HE was diagnosed according to West-Haven criteria  - At inclusion, every patient was examined to rule out overt HE or diagnose HE grade 1  - PHES was performed in all pts. and interpretation was done according to German norms: a score <−4 and/or the presence of HE grade 1 was diagnostic for covert HE  - All pts. were followed-up every 6 months in the outpatient clinic or during unplanned hospitalizations and examined for overt HE  - Median follow-up = 17 months | Prospective, cohort, single-centered, March 2017 - December 2019 | - 65 (27.1%)  - mean age 63 y  - BMI > 30 kg/m² in 40%  - CP B/C = 36.9%  - median MELD = 9  - median HbA1c = 6.75%  - history of overt HE = 16.9% | - 175 (72.9%)  mean age 58 y  - BMI > 30 kg/m² in 27.4%  - CP B/C = 42.3%  - median MELD = 10  - history of overt HE = 13.7% | - 12 pts. were lost to follow-up  - Previous overt HE during the last 6 weeks (stable pts. with overt HE earlier than 6 weeks before enrolment were included if they were under treatment with lactulose and/or rifaximin)  - Chronic alcohol intake during the last 3 months  - Any intake of psychotropic drugs or opioids  - Pre-terminal comorbidities (heart disease HYHA III-IV, COPD Gold C, D, acute or chronic renal failure with creatinine >1.5 mg/dl)  - HCC or other active malignancies  - History of TIPS  - Neurological comorbidities (dementia, Wernicke encephalopathy, stroke)  - Electrolyte disorders (serum K <3.5 or >5 mg/dl, serum Na <130 or >150 mg/dl) | - 33.3% of pts. had covert HE at inclusion and 18.4% developed at least one episode of overt HE (≥ grade 2) during follow-up  - Overall DM was significantly associated with covert HE (OR=2.15, 95% CI: 1.47-3.13; p=0.005)  - Pts. with DM and HbA1c ≥6.5% had increased risk of covert and overt HE compared to non-DM pts. (OR=2.26, 95% CI: 1.00-5.11, p=0.049; p<0.029 respectively)  - DM and HbA1c <6.5% showed no association with covert or overt HE (p=0.233; p=0.174 respectively)  - In pts. without history of overt-HE (n=193), DM was significantly associated with first-time overt HE (aHR 3.23, 95% CI: 1.34-7.76; p=0.009) | - IR or other GMDs were not evaluated  - Covert HE was assessed only at inclusion; serial covert HE testing was not performed during the observational period.  - PHES lacks international standardization  - Pts. were not examined regarding the presence of portosystemic shunts or occult blood loss  - Small vessel vascular brain disease was not assessed - could be a confounder for covert HE  - Subgroups like pts. with vs. without rifaximin and/or lactulose use could not be compared due to small sample size |

**Legend**: pts. = patients; HCV = hepatitis C virus; LC = liver cirrhosis; LT = liver transplant; MELD = Model for End-stage Liver Disease score; CP = Child-Pugh; DM = diabetes mellitus; FPG = fasting plasma glucose; HE = hepatic encephalopathy; WAIS-III = Wechsler Adult Intelligence Scale-III; GI = gastrointestinal; TIPS = transjugular intrahepatic portosystemic shunt; IR = insulin resistance; FPI = fasting plasma insulin; HOMA-IR = homeostatic model assessment of insulin resistance; NCT A/B = Number Connection Test A/B; SD = standard deviation; BMI = body mass index; HbA1c = glycosylated hemoglobin ; T2DM = type 2 diabetes mellitus; ICD-9-CM = International Classification of Diseases, Ninth Revision, Clinical Modification; VH = variceal hemorrhage; HCC = hepatocellular carcinoma; RPG = random plasma glucose; NYHA = New York Heart Association; COPD = Chronic Obstructive Pulmonary Disease, PHES = Psychometric Hepatic Encephalopathy Score

* modified Child-Pugh score excluded HE: range 4–12 points (modified Child-Pugh A/B/C = 4-5 points/6-8 points/9-12 points)

**neurological impairment, decreased cognition with age, medication, alcohol consumption, could be a confounder for HE

**References**

1. Sigal, S.H.; Stanca, C.M.; Kontorinis, N.; Bodian, C.; Ryan, E. Diabetes Mellitus Is Associated with Hepatic Encephalopathy in Patients with HCV Cirrhosis. *Am. J. Gastroenterol.* **2006**, *101*, 1490–1496, doi:10.1111/j.1572-0241.2006.00649.x.

2. Butt, Z.; Jadoon, N.A.; Salaria, O.N.; Mushtaq, K.; Riaz, I.B.; Shahzad, A.; Hashmi, A.M.; Sarwar, S. Diabetes Mellitus and Decompensated Cirrhosis: Risk of Hepatic Encephalopathy in Different Age Groups. *J Diabetes* **2013**, *5*, 449–455, doi:10.1111/1753-0407.12067.

3. Elkrief, L.; Chouinard, P.; Bendersky, N.; Hajage, D.; Larroque, B.; Babany, G.; Kutala, B.; Francoz, C.; Boyer, N.; Moreau, R.; et al. Diabetes Mellitus Is an Independent Prognostic Factor for Major Liver-Related Outcomes in Patients with Cirrhosis and Chronic Hepatitis C. *Hepatology* **2014**, *60*, 823–831, doi:10.1002/hep.27228.

4. Jepsen, P.; Watson, H.; Andersen, P.K.; Vilstrup, H. Diabetes as a Risk Factor for Hepatic Encephalopathy in Cirrhosis Patients. *J. Hepatol.* **2015**, *63*, 1133–1138, doi:10.1016/j.jhep.2015.07.007.

5. Liu, F.-C.; Lin, J.-R.; Chen, H.-P.; Tsai, Y.-F.; Yu, H.-P. Prevalence, Predictive Factors, and Survival Outcome of New-Onset Diabetes after Liver Transplantation: A Population-Based Cohort Study. *Medicine (Baltimore)* **2016**, *95*, e3829, doi:10.1097/MD.0000000000003829.

6. Routhu, M.; Safka, V.; Routhu, S.K.; Fejfar, T.; Jirkovsky, V.; Krajina, A.; Cermakova, E.; Hosak, L.; Hulek, P. Observational Cohort Study of Hepatic Encephalopathy after Transjugular Intrahepatic Portosystemic Shunt (TIPS). *Ann Hepatol* **2017**, *16*, 140–148, doi:10.5604/16652681.1226932.

7. Yin, X.; Zhang, F.; Xiao, J.; Wang, Y.; He, Q.; Zhu, H.; Leng, X.; Zou, X.; Zhang, M.; Zhuge, Y. Diabetes Mellitus Increases the Risk of Hepatic Encephalopathy after a Transjugular Intrahepatic Portosystemic Shunt in Cirrhotic Patients. *Eur J Gastroenterol Hepatol* **2019**, *31*, 1264–1269, doi:10.1097/MEG.0000000000001452.

8. Labenz, C.; Nagel, M.; Kremer, W.M.; Hilscher, M.; Schilling, C.A.; Toenges, G.; Kuchen, R.; Schattenberg, J.M.; Galle, P.R.; Wörns, M.-A. Association between Diabetes Mellitus and Hepatic Encephalopathy in Patients with Cirrhosis. *Aliment Pharmacol Ther* **2020**, *52*, 527–536, doi:10.1111/apt.15915.
