# Supplementary material for: Association between Liver Cirrhosis and Diabetes Mellitus: A Review on Hepatic Outcomes
Source: J Clin Med. 2021 Jan 12;10(2):262. doi: 10.3390/jcm10020262 (PMC7827383; doi:10.3390/jcm10020262)
Supplement: Supplementary file 1 [file jcm-10-00262-s001.zip › Table S2 (1).docx]

Table S2. Diabetes mellitus and variceal hemorrhage

| **First author, year, country of first author, reference** | **Population and Selection** | **Aim and Outcome assessment** | **Study design** | **Comparison** | | **Exclusion criteria** | **Main outcomes** | **Bias/Limitations** |
| --- | --- | --- | --- | --- | --- | --- | --- | --- |
|  | | | | **HD** | **Non-HD** |  | | |
| Jeon H.K.  2013  Republic of Korea  [1] | - 246 consecutive pts. with LC  - 195 pts. in final sample  - 164 M, 31 F  - mean age 53 ± 10.2 y  - Child-Pugh A/B/C = 41%/48%/11%  - Pts. had no history of T2DM  - Diagnosis of HD was based on OGTT, FPI, fasting C-peptide, FPG > 125 mg/dl, HbA1c and HOMA-IR  - Hepatogenous impaired glucose tolerance (IGT) was defined as FPG btw. 100 - 125 mg/dl or 120 min glucose level during OGTT btw. 140 - 200 mg/dl | - To evaluate the impact of HD on portal hypertension and VH  - EGD was conducted in all pts. by two expert GI  endoscopists; varices were classified as small (<5 mm) or large (>5 mm)  - VH within 6 months was recorded retrospectively  - HVPG was measured at least three times by an experienced examiner who was blind to clinical data (including OGTT results) | Prospective, cohort, single-centered, July 2007 - December 2009 | - 108 (55.4%)  - 62% had normal FPG, but impaired OGTT  - M:F = 91:17  - mean age 53.6 ± 9.8 (  - CP A/B/C = 31.5%/55.6%/13% | - 87 (44.6%)  - M:F = 73:14  - mean age 52.7 ± 10.4  - CP A/B/C = 52.9%/37.9%/9.2% | - T2DM before diagnosis of LC (n=28)  - Pts. with HCC (n=11) and severe liver failure  (serum bilirubin >85 µmol/L or HE) (n=7)  - Pts. who did not  provide informed consent (n=5)  - 5 cases of VH in pts. with recent alcohol abuse were excluded from analysis of bleeding risk | - 86.7% had glucose intolerance  - HD was correlated with CP score, VH and HVPG (p=0.004,p= 0.002, and p=0.019, respectively)  - Pts. with VH within 6 months exhibited significantly higher glucose levels at 120 min during OGTT compared to pts. without (p=0.042), but there was no difference in FPG  - Incidence of esophageal VH was significantly higher in HD pts. (p=0.001)  - More HD pts. had large varices (32.8% vs. 17.9%)  - Both groups had similar incidence of gastric VH (1.5%) | - Gender heterogeneity  - Given that HD is not included in classification of DM, official diagnostic criteria or guidelines are not available  - Classification of varices did not include high or low risk evaluation  - Other risk factors (besides CP class and alcohol intake) or prophylaxis for VH were not recorded |
|  | | | | **DM** | **Non-DM** |  | | |
| Yang C.H.  2014  Taiwan  [2] | - 146 pts. with LC  - 82 M, 64 F  - mean age 61 y  - CP A/B/C = 52%/27%/21%  - Diagnosis of DM was based on FPG (>126 mg/dl), symptoms of hyperglycemia, RPG ( ≥ 200 mg/dl), and HbA1C ≥ 6.5% | - To elucidate whether DM is an independent risk factor for VH among LC pts.  - Criteria for VH: clinical signs of bleeding from GI tract, endoscopic signs of active or recent bleeding or large varices with red-coloured sign without other bleeding sources  - HbA1c was recorded within 3 months prior to the VH episode; in the non-VH group, an average of three random values was used | Prospective, cohort, single-centered, (February 2013 - May 2013), with retrospective database (January 2008 - January 2013) | - 37 pts. (25%)  - 57% M  - mean BMI (kg/m²) = 25.8  - CP class B/C = 62% | - 109 (75%)  - 56% M  - mean BMI (kg/m²) = 25.4  - CP class B/C = 40% | - Cases with incomplete chart recordings  - Presence of HCC | - DM and CP B/C were independent predictors of VH [(OR=2.99, 95% CI:1.170–7.660, p=0.022), (OR=4.90, 95% CI:1.697–14.162, p=0.003) respectively]   - In the subgroup analysis, DM significantly correlated with VH only in pts. with CP A (p=0.042) and not in pts. with CP B/C (p=0.128)  - Among DM pts., the VH group had worse glycemic control (HBA1c ≥ 7%) (67%) than the non-VH group (36%) (p= 0.081) | - DM pts. were significantly older (p=0.022), had higher ratio of CP B/C (p=0.043), renal insufficiency (p=0.002) and history of VH (p=0.006) |
| Khafaga S.  2015  Egypt  [3] | - 287 pts. with LC were screened  - 60 pts. in final sample (30/30)  - Similar mean age and liver function btw. groups  - DM was diagnosed based on FPG and 2 h post-prandial glucose level > 200 mg/dl | - To determine the effects of DM on presentation, course and mortality of acute VH in pts. with LC  - Criteria for VH: upper GI bleeding confirmed by EGD; source of bleeding was considered variceal in case of (1) active bleeding or signs of recent bleeding from a varix or (2) a single varix without any other potential source of bleeding; EV were graded from I-IV; GV were classified according to Sarin and Kumar, 1989;  - Pts. were defined as having stable vs. unstable course (HE, SBP, upper GI re-bleeding after initial endoscopic treatment, renal impairment) | Prospective, case-control,  October 2013 - June 2014 | - 30 (50%)  - mean age 53.8±10.5 y,  - 60% M | - 30 (50%) controls  - mean age 52.1±11.9 y  - 80% M | - Age < 18y  - Severely decompensated pts.  - HCC with bleeding or other non-variceal bleeding  - Presence of HE  - Refusal to participate in the study | - Diabetics had more unstable course (73.3% vs. 36.6%) and many hospital admissions during follow-up (1.6 vs. 0.7)  - Diabetics had higher incidence of variceal re-bleeding (46.4% vs. 10%) and higher mortality rate (16.6% vs. 6.7%)  - HE was present in 36.7% diabetics vs. 10% controls | - Small sample of pts.  - IR was not evaluated  - No correlation btw. glycemic control and VH  - The significantly impaired consciousness level in diabetics  compared to controls may be attributed to higher blood loss  - Hepatomegaly in diabetics with LC and DM could be attributed to fatty liver |

**Legend:** pts. = patients; LC = liver cirrhosis; M = male; F = female; CP = Child-PughT2DM = type 2 diabetes mellitus; HD = hepatogenous diabetes; OGTT = oral glucose tolerance test; FPI = fasting plasma insulin; HbA1c = glycosylated hemoglobin; HOMA-IR = homeostatic model assessment of insulin resistance; FPG = fasting plasma glucose; VH = variceal hemorrhage; EGD = esophagogastroduodenoscopy; GI = gastro-intestinal; HVPG = hepatic venous pressure gradient; IR = insulin resistance; HCC = hepatocellular carcinoma; HE = hepatic encephalopathy; HBV = hepatitis B virus; HCV = hepatitis C virus; DM = diabetes mellitus; RPG = random plasma glucose; GI = gastrointestinal;; SBP = spontaneous bacterial peritonitis; GMDs = glucose metabolism disorders; EV = esophageal varices; GV = gastric varices

**References**

1. Jeon, H.K.; Kim, M.Y.; Baik, S.K.; Park, H.J.; Choi, H.; Park, S.Y.; Kim, B.R.; Hong, J.H.; Jo, K.W.; Shin, S.Y.; et al. Hepatogenous Diabetes in Cirrhosis Is Related to Portal Pressure and Variceal Hemorrhage. *Dig. Dis. Sci.* **2013**, *58*, 3335–3341, doi:10.1007/s10620-013-2802-y.

2. Yang, C.-H.; Chiu, Y.-C.; Chen, C.-H.; Chen, C.-H.; Tsai, M.-C.; Chuah, S.-K.; Lee, C.-H.; Hu, T.-H.; Hung, C.-H. Diabetes Mellitus Is Associated with Gastroesophageal Variceal Bleeding in Cirrhotic Patients. *Kaohsiung J. Med. Sci.* **2014**, *30*, 515–520, doi:10.1016/j.kjms.2014.06.002.

3. Khafaga, S.; Khalil, K.; Mohamed, A.; Miada, M.; Mahmoud, S.; Mohammad, M. Acute Variceal Bleeding in Patients with Liver Cirrhosis with and without Diabetes. *Liver Res Open J* **2015**, *1*, 14–20, doi:10.17140/LROJ-1-103.
